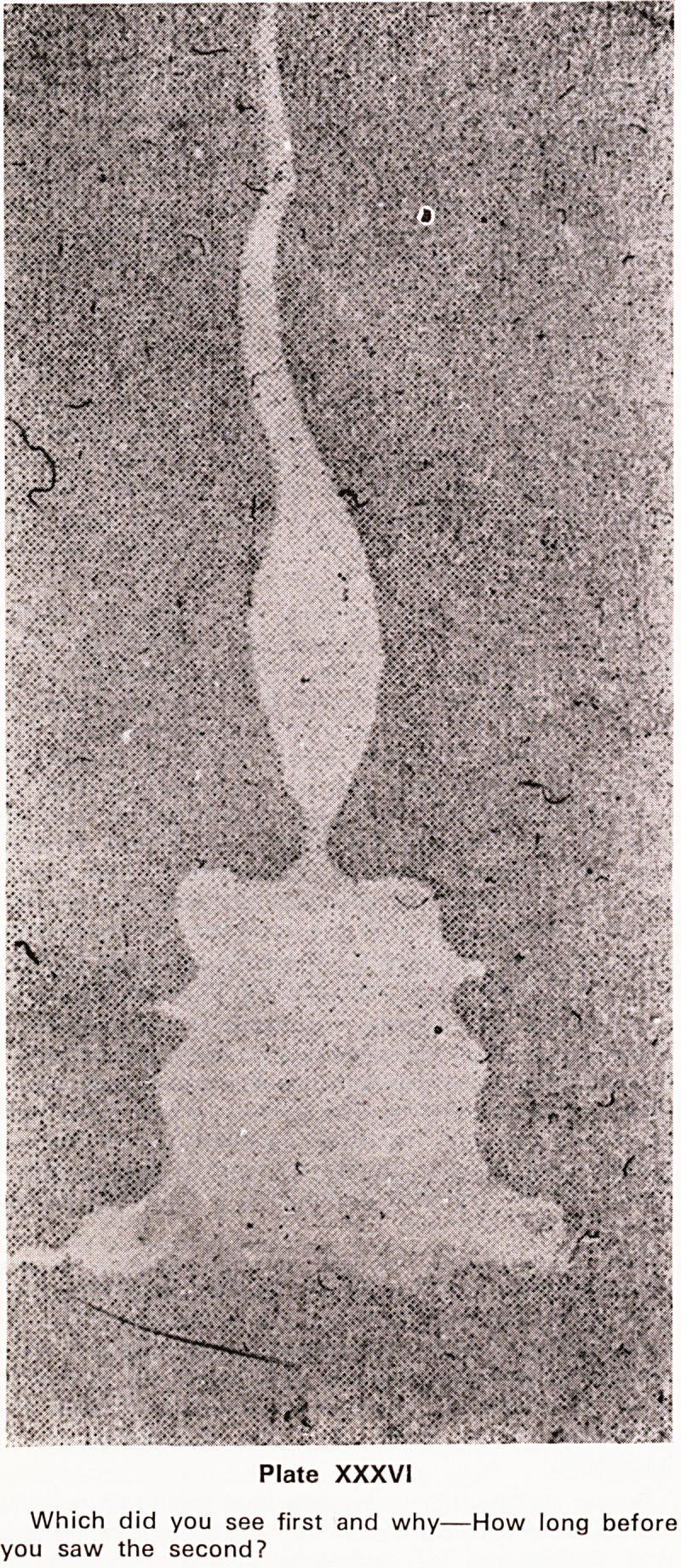# The History of Psychiatry in Bristol
*Presidental Address to the Bristol Medico-Chirurgical Society October 1973.


**Published:** 1974-07

**Authors:** R. F. Barbour

**Affiliations:** Consultant Psychiatrist, Bristol


### The History of Psychiatry in Bristol*

By

R. F. Barbour, M.A., F.R.C.P., F.R.C.Psych.

Consultant Psychiatrist, Bristol

I greatly appreciate the honour you have paid me in asking me to be your President this year—a very special honour for, as you know, the Society celebrates its centenary next Spring.

### The Bristol Medico-Chirurgical Society

I have tried to find out how the Society started and in the Old Infirmary records found quite a lot of jottings and minutes, and with Mr. Jones's help at the Library we were able to locate the original minute book. I thought you would be interested to know that we are the *second* Bristol Medical Chirurgical Society, and there was also a Medical Chirurgical Association.

**Dear Sir,**

It is proposed to establish for Bristol and Clifton a New Medical Society, the prominent feature of which will be the development of the Pathological and Clinical resources of the place, but formed on a basis sufficiently broad to include all the departments of Scientific and Practical Medicine.

At the present time the only opportunity afforded to the profession in this neighbourhood for interchange of thought, and for rendering mutual assistance to each other in their work, is that given by three Meetings of the Bath and Bristol Branch of the British Medical Association. The number of Medical Men practising in Bristol and Clifton is 140; the number of beds provided by public charity (exclusive of the Unions) is 448, and the number of Out-patients treated annually at the public hospitals is over 50,000. It is believed that any attempt to utilize these resources is likely to meet with general approval, and to be attended by success similar to that which has been accorded in most large provincial towns where like societies have been established.

You are invited to attend a Meeting at the "Museum and Library," Queen's Road, on Tuesday Evening, the 24th inst., at Eight o'clock, to assist in the formation of such a Society.

We are, dear Sir,

Yours faithfully,

NELSON C. DOBSON, F.R.C.S.,

R. SHINGLETON SMITH, M.D., Lond., B.Sc.,

WM. HY. SPENCER, M.B., Cantab.

Museum and Library,
February 20th, 1874.

**Plate XXXI—The invitation to the first meeting of the Bristol Medico-Chirurgical Society**

The first was founded on the 23rd April, 1812, and was really a Medical Reading Club. It had only 12 members, its purpose being 'friendly intercourse and the circulation of books'. The second, the Association, started some seven years later on 6th May, 1819. It was a dining and discussion club, the members met for dinner at 5.00 p.m. Richard Smith was Secretary—its aim being 'to promote the interests and respectability of the profession'. An item on an early agenda reads 'that both physicians and surgeons should consider themselves on a par with regard to charges'. To begin with the Association met quarterly but I have still to find out when it last met.

Our own Society has met without a break since 25th March, 1874 (Plate XXXI). The first President was Dr. Frederick Brittan (Plate XXXII). Two years later there

**Plate XXXII—Dr. Frederick Brittan, first President of the Society**

* *Presidental Address to the Bristol Medico-Chirurgical Society October 1973.*

was much discussion as to whether the Society should entertain the British Association on its forthcoming visit to Bristol—this was eventually turned down, the Secretary in the minutes adding 'thereby affirming the principle upon which the Society started, viz. (to wit) to restrict its operations to intellectual work'—so without more ado I must turn to the main part of my address.

**Society and its Outcasts**

My particular interest has always been Society and its outcasts and, although to many the mad may seem very different from the bad, yet they have much in common—why cannot Society tolerate them—what are the problems which arise when they are excluded and can we improve on our present methods of dealing with them?

To begin with I want to look at the very big contribution that Bristol has made in helping the 'mad'. From the earliest of days, communities have always reserved the right to decide who was undesirable and what was to happen to them. The odd, the weaklings, the ineffective have always been at risk and I read recently that Eskimo parents still stuff their first-born daughter's mouth with snow and leave her out to die. In this country up to the XVIth century we looked to the Church to care for the very poor, the lepers, the infirm and often the lunatics. Some of you may not know that the word 'bedlam' is derived from Bethlehem as it was the Priory of St. Mary of Bethlehem at Bishopsgate in London, founded in 1247, which took a special interest in lunatics. Records show that six were there in 1403, and in 1547 Henry VIII gave it to the City of London as a Hospital for lunatics.

**Early Madhouses**

Records of the 17th century show that there were a considerable number of private madhouses where the well-to-do could make arrangements for their relatives to be looked after in reasonable comfort. There may have been an Asylum at Kingsdown, Box as far back as 1615, certainly in 1815 when evidence was being given to the Select Parliamentary Committee it was stated that there had been one there for over 200 years.

The position however was very different for those being looked after by the parish. The non-rich misfits tended to be all thrown together, the vagrants, the elderly, the disorderly and the frenzied all those in fact who were not considered to be criminals. John Cary of Bristol, an economist, merchant, social reformer and Quaker, was very concerned about the "non-productiveness" of these outcasts and submitted to the Mayor in 1696 his 'Proposals for the better maintaining and imploying the poor of the city of Bristol'. Apprentices were to be trained, places of work provided for the unemployed and the elderly were to be cared for. To do this he suggested that the 19 parishes in Bristol be grouped together so that conditions could be standardised and it would then be easier to make appropriate arrangements a clear-cut line was, however, to be drawn between those who lacked facilities for work and those who were unwilling to work.

His scheme was quickly adopted. A workhouse called Whitehall adjoining Bridewell was chosen for 100 girls to be trained as wool carders or spinners. The following year the Aldworth family mansion, until recently the Mint was bought for £800 from Colston and was renamed St. Peter's Hospital. It included a work house for 100 boys to weave fustians and calimancoes. A medical service was provided 'we give warrants to our physicians to visit them, such as wanted the assistance of our surgeons were directed to them.' St. Peter's Hospital continued as a Hospital till 1861. For 80 years after that it was the administrative centre of the Board of Guardians, the Public Assistance, many of whose functions are now exercised by the Social Services.

Planning for other people is a dangerous occupation unless they see the problem as you do. It is easy to evoke a strong emotional reaction, none of us like to be thought of as an 'under-dog', it is a slur on our capabilities and one way we can show this is to denigrate the provisions made. The words work-house, reformatory, approved school, thought up with such care become offensive—equally, the insane have been put into receptacles, madhouses, or in asylums or retreats while the gap between us and them is stressed by calling the doctors, who care for them, alienists.

**Plate XXXIII—St. Peter's Hospital in flames during an air raid in 1940. (Reproduced by permission of Reece Winstone Esq.)**

Psychiatrists may call themselves mental health specialists but one can still, at times, detect a certain emotional reaction to the term—'I don't need to see one of those—I am not as bad as all that'.

St. Peter's Hospital will be remembered by many of you as a building of outstanding architectural beauty. Unfortunately it was blitzed in November, 1940 (Plate XXXIII). The Infirmary had been opened in 1737 and for over a century the two were rival medical institutions, with many of Bristol's best known physicians giving their services at both places. St. Peter's cared not only for the infirm and all the other poor but it also looked after the 'frenzied' and was the first provincial lunatic hospital. A year after its opening there is reference to 'Widow Sweet', a crazy woman who was taken in so that she might be prevented from squandering her estate'. In 1827 there were three wards for lunatics.

The big step forward made by Bristol in setting up the Incorporation of the Poor was the sorting out of the different types of 'non-effective' and 'non-productive', and providing some degree of care for those who could not pay.

Of the early private 'madhouses' possibly one of the better known was that run by the family of Mason and Cox. One of our colleagues, Temple Phillips, submitted this summer a thesis on this well known private madhouse—one of the largest outside London. Originally in Wickwar, Mason moved to the Fishponds area in 1740 and an early map shows Mason's Madhouse. Five generations of the same family cared for the insane but the best known was Joseph Mason-Cox. Cox was probably the first regularly qualified physician who studied medicine in order to specialise in mental disease. He graduated M.D. in 1787 with a thesis entitled De Mania. In 1804 he published 'Practical Observations on Insanity' and for the next forty years it was considered to be one of the best text-books on the subject. It was translated into French and German. Cox's book in discussing treatment illustrates the alternatives that physicians then found themselves with and which in a way still affects us today. He divides the various means to be adopted in attempting the cure of insanity into moral and medical and under moral he places management. 'The essence of management results from experience, address and the natural endowments of the practitioner and turns principally on making impressions on the senses' (p.25). He continues that the aim of the physician is 'to procure the confidence of the patient or excite fear. The first (confidence) may be obtained by very varied means: thus I have seen the most furious maniacs being liberated from their shackles by my direction, and under my own immediate inspection became so attached and devoted to me as never again to require coercion. Fear must be excited by firmness and menaces, by strong impressions on both mind and body while confidence often results from soothing and gentleness and I am decidedly of opinion from much observation and experience that more is to be gained by these last than by their opposites.'

The fact that many mentally ill people seem to be out of touch with what others consider the real world has led to a variety of treatments whose primary aim is either to affect the senses or the emotions. Erasmus Darwin believed that all diseases arose out of disordered motions of the nervous tissues of the body but it was Cox who popularised the 'swinging chair'—like a rocking chair it could be soothing or like the big wheel at the fair it could be highly disturbing—quoting from his book 'a variety of means might be adopted to excite a new order of symptoms creating considerable commotion in the animal economy interrupting the morbid association and even occasioning temporary disease'. Incidentally he was also a great believer in the effectiveness of music in the treatment of psychotics.

Later generations of psychiatrists have tried the effect of the continuous bath which, again, by altering the temperature of the water can be made soothing or stimulating. Electric currents have been used to provide sleep as well as convulsions.

In Bristol in the 19th century there were several other private establishments, a small one, Whitehall House, St. George, on the North side of the Turnpike Road, supervised by J. Braithwaite Taylor, then there was Rudgeway Manor under the supervision of Nehemiah Duck. Dr. Longworthy at Long Ashton took lunatics into Longwood House. Better known, however, was Cleeve Hill probably the same as the Hanham Home spoken of so highly by Wesley. In his Journal of Saturday, 29th September, 1781, he wrote, 'I spent an hour with Henderson at Hanham and particularly enquired into his whole method: and I am persuaded there is not such another house for lunatics in the three Kingdoms. He has a peculiar art of governing his patients not by fear but by love—the consequence is that many of them speedily recover and love him ever after.'

**Brislington House and the Foxes**

But I want to turn now to the other specially built private madhouse and the family who devoted their life to the welfare of the insane, Brislington House and the Foxes. From 1804 to 1951 Brislington House (Plate XXXIV) was one of the best known private mental homes in the Kingdom, now of course it is the Nurses Home for the United Bristol Hospitals.

Edward Long Fox (the elder) well illustrates the all round creative ability of the early physicians of

**Plate XXXIV—Brislington House**

Bristol. Born 26th April, 1761, a Quaker, he came up from Plymouth where his father was a doctor. He was physician at both St. Peter's and the Royal Infirmary (1784-1816). In 1794 he took over from Henderson at Cleeve Hill. He invented the Rumble, a small square carriage on two wheels (rather like a hackney) with a seat on the roof for the driver. He bought Knightstone Island at Weston-super-Mare and set up there Public Baths. He was commanded to attend on George III at Windsor during one of the King's illnesses but declined to take the case on. Brislington House was opened in 1806 at a cost of £35,000—he built it despite considerable local opposition.

Fox realised how 'sensitive' many insane patients are, further how much they disturb their relatives so that making a break becomes essential. Removing them from home and placing them in an asylum, a refuge, is often the first step towards their recovery. Of Brislington House he wrote 'it is a receptacle for lunatics as commodious as possible for the benefit and safety of the patient'. The House consisted really of eight units—connected at basement level—three each side of centre, on the one side for the women, on the other for the men, with a fourth house at each end for the physically sick. The patients were allocated according to sex, according to social status and financial resources, and according to severity of illness. Each block had its own quadrangle for exercise at the back. This, as he said, allowed patients of similar social background to be accommodated together. There were five courts, greyhounds were kept and he provided many other facilities for recreation or occupation.

Fox well appreciated the importance of religion and on Sunday, although a Quaker, he provided opportunities for Divine Service but kept the sexes segregated. For a few he even provided separate houses on the estate where they could be looked after by their own servants. In 1814 there were 70 patients, 28 servants. Parry Jones in his book, The Trade in Lunacy, p.112, says of Brislington House, 'This establishment was one of the most reputable provincial licensed houses in the early nineteenth century and was undoubtedly the finest of the small number of purpose built houses'. Brislington House was so successful that Henry Hawes Fox who followed his father at the Infirmary built Northwoods near Winterbourne in 1833. Although Brislington and Northwoods were both managed by members of the same family, the standards were very different and considerable family acrimony ensued. In 1853, J. G. Davey took it over. He was an early president of our Society and addressed our predecessors on 'materialists versus metaphysicians'. He objected to terms such as Life—will—mind —preferring concrete terms like cerebration.

One of Fox's staff from Cleeve Hill, Katherine Allen, went to Tuke at The Retreat at York as their first Matron. Tuke, writing on the 'moral influences on the insane' said 'the experiment was sufficiently wide and general to prove not merely the impropriety of using chairs and whips in the management of the insane but also the almost infinite powers of judicious kindness and sympathy on disordered minds and consequently the extensive applicability of moral agency in the management and curative treatment of insanity'.

The Commissioners in Lunacy in their report in 1857 write 'The Moral Treatment of Insanity comprehends all those means which, by operating on the feelings and habits, exert a salutary influence and tend to restore them to a sound and natural state'.

Association with words change, particularly so when the subject is one about which there are strong feelings—such a word is Moral, it had originally both an emotional and ethical denotation, now we tend to use it less in the sense of the mores or customs but rather as a standard which a person should live up to.

In 1778 James Vere—a London Merchant and a governor of Bethlem Hospital, wrote a book entitled 'A physical and moral enquiry into the causes of the Internal restlessness and disorder in men'. Vere pointed out the conflict that occurs between the lower order of instincts and the moral instincts (p.466), 'thus creating a sort of internal war, which divides the man against himself: and hence a large share of disquiet and restlessness will be the unavoidable consequence'. This, of course, is nothing new, St. Paul in his Epistle to the Romans (Chapter VII, 23) wrote: 'I see another law in my members, warring against the law of my mind'. I believe Socrates considered every foolish or vicious person as insane or morally mad but it was a Bristol Physician, James Prichard, who was the first English psychiatrist, in his 'Treatise on Insanity' (1835, pp.12, 21, 23) to describe a new group of mental disorders which he called 'moral insanity'. He describes them as a 'morbid perversion of the feelings, affection and active powers without any illusion or erroneous convictions impressed upon the understanding'. Later he writes 'one of the most striking of these forms is distinguished by an unusual prevalence of angry and malicious feelings which arise without provocation or any of the ordinary incitements', and again 'a propensity to theft is often a feature of moral insanity and sometimes it is its leading if not the sole characteristic'.

**James Prichard**

James Cowles Prichard, born in 1786, was elected Physician to St. Peter's Hospital in 1812 and to Bristol Infirmary four years later. He was a Quaker from Ross-on-Wye who, after attending St. Thomas's Hospital in London went to Edinburgh and there started his work on the 'Researches into the Physical History of Man'. He was a man of wide interests, ethnologist, anthropologist and philologist. In Bristol he lectured on Egyptian Antiquities and in 1819 he published his 'analysis of Egyptian Mythology'. (Whether our past President will agree with his theory of 'the Eastern origin of the Celtic Nations', I do not know.) He lived in the Red Lodge and became one of the First Commissioners in Lunacy in 1845.

In Bristol this year there was formed the Prichard Society, a meeting of the legal and medical professions for it is as a Forensic Psychiatrist that Prichard will be remembered. By setting up the concept of Moral Insanity Prichard highlighted a problem which remains with us. Psychology and Sociology are always trying to find terms which can be used non-judgementally, at present the word, stress, originally chosen to indicate an imbalance—no growth can take place without stress, is becoming something to be avoided. The concept of Moral Insanity bothered people, even more so Moral Imbecility, could you be born without a sense of right or wrong—so the term Psychopath was introduced, that in turn became a term of abuse—'he is a psychopath' implying hopeless, irredeemable, and Social Deviant is at present the term of choice.

### Thomas Beddoes

A Fourth Bristol physician whom I must mention is Thomas Lovell Beddoes (1760-1808). Beddoes came to Clifton in 1793 from Oxford where he had been Reader in Chemistry. He was very interested in the possibility of pneumatic medicine for curing diseases such as chlorosis, hypochondriasis, dyspepsia, scurvy, asthma, dropsy and hydrothorax. In 1798 at 6, Dowry Square, he opened the Pneumatic Institute where patients could inhale factitious airs. Some of you may have seen the B.B.C. Telecast earlier this year on the Pneumatic Institute. Humphry Davy was introduced to him by James Watt and became his Research Assistant. The effect of gases particularly oxygen and azotic gas or nitrous oxide were fully explored. Beddoes describes the effect of nitrous oxide as like being bathed all over with a bucket of good humour when a placid feeling pervades one's whole frame.

In his Essays published under the title of Hygeia, there are many references to insanity, to the effects of the mind on physical disease and of the passions on the body. 'The sane and insane mind are made up of the same stuff. A change in hues and arrangement of their materials is the sole difference. Upon the knowledge of this change it is probable that the power of preventing and correcting it greatly depends. The management of the insane as far as one can judge from books, goes on too much in the gross; and without the insight to be obtained from the study I recommend, it is not easy to say how it can be more nicely adapted to the exigencies of individuals. The consideration of moral causes of slow operation is the most curious as well as most useful in this whole enquiry'. Beddoes was particularly concerned in the prevention of disease, in fact he renamed his Pneumatic Institute the Preventive Institution.

### The Burdens

In 1895 Harold Burden came to Bristol and was Chaplain at Horfield Prison. His wife had worked with Octavia Hill. They were very aware of a constellation of factors, dullness—alcohol—poverty. The Royal Victoria Home was opened in 1899 for alcoholic women and girls in moral danger. In 1902 they founded the corporation known as the 'National Institution for persons requiring care and control' and in 1903 opened up the 'Brentry Certified Inebriate Reformatory'. They bought Stoke House, the Dower House of the Beauforts, which was the original building for what we now know as Stoke Park Hospital. The Burdens were farsighted people and provided money for the Burden Mental Research Trust where the many facets of mental ill health have been studied by animal researchers, geneticists, endocrinologists, neurophysiologists, clinicians, surgeons, each making their essential contribution.

| Name and Age. | Crime. | When and where tried. | Remarks by judge. | Whether before convicted. | Time supposed to have lived on crime. | Character of Parents. | Remarks by Chaplain. | Remarks. |
|---|---|---|---|---|---|---|---|---|
| John Nicholls aged 7. | Stealing monies. | June 12, 1846, at Warwick. | None (!) | Not known. | Not known. | Bad: Connections bad. | None. | Sent to House of Correction, under the provisions of a conditional pardon. |
| Dominick Rafferty, aged 8. | Stealing 9d in copper. | Oct. 21, 1846, at Preston. | "Must be separated from his family and connections." | Not known. | Lived by crime, from the time he was capable of committing it. | Thieves and vagabonds; father tramps about the country; an elder brother transported; another 12 years old who has been eight times in prison. | "The habits and society in which this poor child has lived, render it almost impossible to develop, in his short stay here, any good tendency in his feelings or disposition. | Rejected by order of the Secretary of State, as unfit to be received. |
| These two children were believed, from their appearance, to be little more than six years old. | | | | | | | | |

**Plate XXXV—Details of two children held at Millbank Prison for transportation**

### The Care of Misfits

Let us look briefly at another aspect of helping social misfits. I mentioned that St. Peter's in 1697 had an Apprentice School for Boys—it was not, however, till much later that the needs of the children were more adequately met. Muller's Orphanage started in 1836 but in 1850 young children were still being transported to the Colonies.

Plate XXXV shows details of two children held at Millbank Prison under sentence of seven years' transportation.

Mary Carpenter published in 1851 her book on 'Reformatory Schools for the children of the Perishing and Dangerous classes and for juvenile offenders'.

In 1852 she established the Kingswood Reformatory for boys, two years later the Red Lodge, Prichard's old house, was opened as a Reformatory for Girls; in 1856 there was passed the Industrial Schools Act and during the second half of the nineteenth century at least five industrial Schools were set up in the Bristol area.

During the first half of the nineteenth century the lack of adequate accommodation for pauper patients became a scandal. Conditions in most of the Private Mad Houses left much to be desired. In 1828 there were only 12 county asylums.

### Glenside Hospital

St. Peter's was ill arranged, jail-like, depressing, grossly overcrowded without exercise and airing grounds. To bring in a topical note Cholera raged there in 1832. Eventually a New Institution, the Bristol Borough Lunatic Asylum, was opened at Stapleton. On 27th February, 1861, 50 male and 63 female patients were moved to Fishponds. However, four years later the Commissioners were deprecating the overcrowding 'each dormitory had *one* bed too many'.

The next meeting of the Society is to be held at Glenside Hospital and it may be more appropriate to refer then to some of the changes that have taken place but tonight I want to mention two. First, Industrial Therapy set up in 1958, thanks to the initiative and enthusiasm of Donal Early. The previous year the Commissioners highlighted the problem of the long-stay patients and stressed 'that the aim of modern in-patient care should be no longer custodial but curative and directed towards returning the patient to the community'. The importance of satisfying work, preferably outside the Hospital grounds, seen as a necessity by Cary had also been noted by Fox and is still regarded as an important factor in maintaining Mental Health. Then if work is important so too is leisure time and the opportunity to feel one's feet in the outside world.

Unfortunately many patients do not have relatives who can look after them and many remain on wards as nothing else is available for them. Hostels are usually for short stay so gradually, starting in 1965, houses have been acquired where people from Glenside can go and live but where it is easy to arrange professional supervision. I believe there is now accommodation for nearly one hundred ex-patients who need some extra care.

My colleagues always keep me up-to-date and since writing the above I have come across Donal Early's article in a number of the *British Medical Journal* (1973) published last month describing the Bristol Industrial Therapy Housing Association and I want to refer to it again later.

**Recent Developments**

Barrow Hospital, with its well-separated and diversified villas, is a relatively recent development being opened in 1938. It may be an administrative headache —I am told that one Matron used a motor-scooter to do her rounds—but it preserves, at any rate from the windows, a slightly more normal environment for patients. Again, note the Day Hospital opened in 1951 in Clifton giving not only relatives some relief but affording patients help and specialised care during the day yet allowing them to return to their family at night. It was the first day hospital in Great Britain outside London. The Day Hospital, I.T.O. and BITHA are all forms of care, half-way between full hospitalisation and looking after yourself whole-time at home.

Developments in Bristol are well known to most of you, the Establishment of a Chair of Mental Health, wards in the general hospital, medical students being required to take mental health as a subject in the Final Examinations, are all evidence of the increasing awareness by the Community of the importance of mental health.

**Lessons from the past**

I want now to use this brief account of Bristol and its lunatics to focus on how society seems to deal with its misfits and then see if there are any lessons for us at the present time.

My friends and some post-graduates occasionally refer to Barbour's Seven-League Boots—I start off with some fact—dive like a porpoise and surface leagues away, the connecting link clear to me but not always to them. I will do my best to avoid this trait tonight.

First we had Cary sub-dividing a group, lets call them the unproductive or 'Work-less'—into the young and untrained—the unlucky but employable, the physically handicapped, also the aged and the frenzied, for this group it was to be care and good trade facilities, but there were also the vagrants and those who would not work—for them unless they mended their ways it was to be prison—they were to be made more uncomfortable. The advantages of consolidating the parishes were obvious, a greater degree of specialisation based on a larger group at risk and more uniformity of care. The speed with which other towns copied Bristol showed that the scheme met a real need.

**Fox** at Brislington House used the same principles of differentiation and specialisation and carried them a little further but for a particular group—chiefly the rich—though he did have a few paupers—for him the factors were sex, social status and severity of illness. He lessened strain by removing them from their relatives and placing them under the care of skilled and experienced people. He avoided increasing stress by not mixing the social standards, keeping them with their peer groups. He saw his patients as individuals needing specialist help.

**Burden** worked along the same lines—subdividing his group and seeing how differing disabilities affected each other. He isolated the inebriates, people who had tried to alter their sensory field, in particular their discomfort and anxiety, usually by taking alcohol. He also focused on the various kinds of mental defectives who were being increasingly seen as a separate group from the mentally ill.

**Beddoes** with his factitious airs and pneumatic institute may seem to us a rather strange physician with some unusual theories and he certainly was not very popular with his colleagues, though this, in fact, may have been as much on account of his political views as his original medical ideas. Preventive medicine was his primary aim and he realised the importance of early patterning. **Prichard** and **Mary Carpenter** in very different ways were concerned with social adjustment and moral management but both recognised the group of acting-out individuals who did not wish to or possibly could not, conform to the standards expected of them.

**Fox** and **Carpenter** both stressed the importance of the personality of the care-taking person. Our medical training involves us in techniques which by their very nature are hurtful and our training is such that we have to consider our patients as objects rather than people—things into which we can stick needles or cut open without undue discomfort to ourselves and most of us have become very good at it thanks to the repressive effect of habit.

I would suggest that our work is far more affected by our own personality and disposition than we usually realise. A too distant—too objective an attitude may not be adequately reassuring to the troubled patient. A good bedside manner is the result of three things—a caring attitude by the physician—the ability to create a feeling of confidence and the ability of the patient to accept, but the warmth must get through. In our present phase of scientific medicine I think we underestimate the importance in illness and disease of the physician and his personality and of course under this term, physician, I include all the clinical specialties, it may well be that once the pre-operative diagnosis has been made the personality of the anaesthetist is more important to the patient than that of the surgeon.

**Failure to conform**

But to return to Psychiatry. It is still the specialty with the biggest number of beds, in fact 47% of all beds in the South-West Region are allocated to Mental Illness and Mental Handicap and the average daily number of beds supervised by psychiatrists last year was 14,522. We know the proffered symptoms are changing and we do not always know why—possibly the huge increase in the prescription of drugs acting on the central nervous system means that some conditions are caught at an early stage.

The other half of the social misfits are found in the prisons, predominantly male, cared for by Governors and until recently within a very hierarchical structure. In the South-West Region there are about 3,200 prisoners in Borstal or prison, you could probably double the number if you add those with suspended sentences or on parole, say 7,000.

I have given two sets of figures but the two populations overlap—20% of those in Mental Hospitals have been in prison and 20% of those in prison have been in psychiatric units. The thoughtless, those taking a chance, the inadequate, get sent to both types of place.

How come that such a large proportion of the community are failing to conform?

From the moment of conception, an entity is growing in an environment, there is continuous INTAKE and OUTPUT and we are slowly learning how maternal output, once it becomes foetal intake, modifies foetal structure. With birth, the environment is completely altered but there is still this process of intake—heat—oxygen—light—food, all affecting development.

Study of infants in the last decade has focused attention on facts such as how many times a mother speaks to her baby or picks him up and gives him her full attention or whether a mother enjoys her baby, whether the baby comes up to her expectations. In Bristol there has been a special study of what is called prop-feeding where the infant sits sucking from a bottle that is propped up, and remains with eyes focused on one spot. Do you remember the recent B.B.C. television programme on Lorenz and the geese, the human Foster Mother picking up the eggs, speaking to them—Vi Vi—and the colouring of her boots so that a pattern was there that could be imprinted when the central nervous system of the gosling was ready to respond. We are already beginning to find out that the frequency and intensity of stimulation will affect the response.

At certain stages of development reflexes are conditioned, that is circuits completed. 'Imprinting' is a term from animal research, conditioned reflexes from physiological experiments, electric circuits from the neurophysical laboratory. Before mentioning John Bowlby I would like to quote Thos. Beddoes again who wrote 'Biology, the doctrine of the living system in all its states appears to be the foundation of ethics and pneumatology' the term he used in preference to psychology.

John Bowlby writes 'Even by the time the second birthday is reached the prefrontal lobes remain very little developed. These parts of the brain, evidence suggests, are necessary if an individual is to inhibit immediate response so that a plan of action, dependent on factors not present in the immediate environment can be carried to completion. Consistent with that, it is found that only towards the end of the pre-school years are most children able to make a choice that gives substantial weight to factors not present in the here and now. '..............' without the necessary neural equipment, behavioural equipment cannot be elaborated; and until it is elaborated, behaviour remains more in keeping with the pleasure principle than the reality principle'. This is very reminiscent of St. Paul with his law of the body in conflict with the law of the mind.

Imprinting in animals is probably not greatly different from attachment behaviour in man. This whole area of development is a fascinating one with much learned from feed-back systems, self-correcting like a homing missile, although in human terms we prefer to say Adaptation or Adjustment to Society.

The selection of astronauts is teaching us a great deal about sensory change and deprivation. Few men brought up on our earth have a central nervous system which can adapt to weightlessness or constant sound and yet still allow them to carry out routine tasks, most when sensory input is drastically reduced either panic or act irrationally, that is as if mad. This reaction to reduced stimulation is comparable to the increased stimulation which is the common factor in most types of brain-washing. When in a situation where personal independence is valued, it is comfortable to overlook our own marked dependence on our environment.

Returning to the toddler, there comes a time when a constellation of stimuli forms a gestalt, an entity, a thing, a person, which has characteristics of mobility which has much power over us as it can meet our needs, refuse them or make us wait. To be considered 'good' by them has often to take precedence over feeling good internally. We learn the difference between immediate satisfaction and delayed reward and we develop a feeling about ourselves and our environment—that it is dominating, that we are evenly matched, that it is inferior. A comparative relationship has been established, though the actual outcome may well vary with the differing of the two parties' strengths at different times (Fig. 1). As we grow we are trained, confined, praised, punished and we become expert in reading cues, in anticipating what will be required of us, this continuous feed-back modifies our reactions and gradually our personality emerges as we develop habits of coping with the demands and expectations of our environment. All this is equally true of Saints as well as Sinners. Saints, Deviants, Normal people all find themselves at times in conflict with the community they live in, that is provided they have

**Fig. 1**

enough ego drive to struggle, some may have succumbed earlier.

Each community varies its standards according to its needs, one culture's food is another's poison. When a society feels threatened it closes ranks and throws out the doubtful, white feathers for conscientious objectors, dubbing pilots as having low moral fibre, a descriptive term, neither cowardice nor an anxiety neurosis but definitely derogatory.

Sociologists would divide non-conformers into three big groups.

1. Those who understand the aims but do not agree.
2. Those who object to the means rather than to the ends.
3. Those whose upbringing was such that the mere fact of being told to do something evokes a negative response.

Whether we are an active non-conformer or a passive resister or a withdrawer into our shell will depend on what has happened previously between us and the caretakers, who in our culture are usually our mothers, not even the extended family.

The layman would probably categorise non-conformers according to their behaviour, the dangerous criminals, the social nuisances, for instance persistent traffic offenders and alcoholics, the socially inadequate, tramps and drop-outs, the worriers, the depressed and the real mad—a continuous spectrum, which group anyone is most bothered about probably depends upon their own social class and professional work. There is, however, at this time again a clash, as in John Cary's time, between the tax-payer and the unproductive, why should they get away with it—should food be provided for those who cannot be bothered with the rat race? Mary Carpenter in 1851 comments on those 'who will never adapt voluntarily'. Those coming from 'close noisome dens which present nothing revolting to their feelings, they prefer them to a clean abode where they must resign their occupation and some portion of their liberty'.

The problem of non-conforming seems to stem from the stage of personality development when a child (say before three years old), finds itself clashing with another individual, child or adult when our fore-brain has not yet taken over. The tension arising from interpersonal confrontation has been experienced by each and all of us and each of us has his or her own way of dealing with it. Anticipation of a conflict tends in itself to increase the tension. The pattern of 'something is expected of me' continues throughout life—though what is expected will vary—it may be 'say Please', later asking permission, at work letting someone know you will be out of the office. If no socially acceptable solution is found, the tension may build up until our bodily functions are altered or we do something which forces those around us to act, an overdose, shoplifting or the like, sometimes we may feel its just not worth it and walk away.

It is these situational clashes which makes the sociologist doubtful about the medical analogy. In a clash between bacteria and a person we build up the patient's resistance or give them a bactericidal drug, in a sense the bacteria is always wrong. I am reminded of the story attributed to the Edinburgh lunatic, 'I said the world was mad, they said I was mad, and confound it they outvoted me'.

Is it the individual or is it the Society that is off course?

It is interesting to see how unofficial groups like the Samaritans have caught on. They are available at any time, the interview does not start off with 'name and address please'. You go to them of your own choice and they have only the power that you allow them. You meet on your terms not those of the establishment.

Returning to our deviants, at the one end we have the violently explosive, the intensely egocentric who expect to get away with it and at the other the tramp, the 'drop-out' who, as Mary Carpenter says, are content with their lot, who in our time and age do not see much point in profits and material competition, who question the need for regular hard work; but who admittedly function like myself when they see a situation as a crisis but see no point in being stretched 365 days in the year, after all as one of them put it to me, 'you can't wear more than one pair of shoes at a time so two pairs is surely ample'.

Inevitably this raises two points, Social Welfare and Conscience. If by doing some very disagreeable but highly paid job you earn enough in six weeks to support yourself for six months on Social security, is that essentially different from the professional or business man who also makes enough in six weeks or possibly in six hours to support himself for six months, though I grant you he does not usually draw unemployment benefit.

But what about conscience, that sense of botheration or internal disquiet. It has a quantitative and a qualitative side. By quality I mean the acts or non-acts which evoke this particular feeling—here all one can say is that there is no general agreement among the human race as to what is right and what is wrong. Behaviour chosen by one culture is abhorrent to another. The quantitative side, the intensity of the feeling would seem to be brain determined. Alter the circuits leading to and from the fore-brain and right and wrong become an academic matter rather than a question of guilty action. The Church has long recognised a 'scrupulous' conscience, that is one which cannot accept confession and absolution. Is Prichard right, is there at the other end a conscience-less person? All one can say is that our electro-encephalographers have not isolated any brain pattern common to all serious criminals.

Both the psychiatric and the penal world are very concerned with what happens to someone when he is put away against his wishes, especially if he lacks any true insight. Locks and bars, drugs, high staff-client ratio, all have the same aim, to keep the individual in the place which Society has decided is the best place for him. It then becomes a question of Moral Management or education and training as we would term it.

What are to be the reforming influences, if placement in neutral, non-judgmental surroundings, a retreat, is not enough, how are the early patterns to be changed, so that a more conforming person emerges. Is it to be loss of liberty—aloneness—boredom—in the medical world everything is done to counter that. We try by our care and interest to convince the client, patient, that they do really matter. Autobiographies of recovered schizophrenics show how much they were aware of what was happenening around them even when they did not respond. Do we aim at self-assessment aided by comparison with similarly afflicted people, the continuous comparison between self and others that goes on in every hospital ward or floor in a penal institution. Most patients have given up the fight, and we feel they need to fight more, most prisoners have acted out too much and we think need to be quelled. The gap between the forms of treatment of the mad and of the bad is narrowing. Positive reconditioning is in vogue that is exposure to something to which you are irrationally attached, alcohol, pornographic pictures and receiving at the same time an unpleasant stimulus. How many of you saw the film 'The Clockwork Orange'? Much is still unclear, it is only since writing this paper that I have really thought through what is involved in a judicial sentence with, provided you conform, a known date of release, versus the indefinite state of the certified patient, or of the lifer, who to win release has either to recover or conceal his symptoms.

If the crucial problem is the Rights of the Individual and the Rights of Society and if many of the problems arise because the individual's sense of His Rights and their Right has gelled in a comparative form suitable for an early toddler's life but not for later adult life, possibly the only curative measures lie in regression to infant-like states—where sensory impressions are all important, where behaviour is emotionally rather than intellectually determined, something that is only possible in individual, one-to-one, therapy or in peer groups where people are allowed to speak their minds and act out their feelings. Many of you may be uncertain about this, possibly it is too threatening, we are after all, compared to others, a repressed culture, but let us return to Earth and Bristol and one very real need at present, more half-way houses, whether from psychiatric or penal institutions. Mary Carpenter wrote 'it is only in proportion as there is liberty that security can be felt in the child's improvement'. If a person remains away from his social group for a long time he or she needs help when they return. At the end of the war I was one of a team concerned with the resettlement of returned prisoners of war. It had been their patriotic duty to be as obtuse, annoying and as difficult as possible to their captors, now for their own sake we wanted them to conform quickly to our wartime British pattern. The ease with which they could readapt was clearly related to their pre-war personality pattern.

Everyone agrees to patients and prisoners being helped but please do not set up a home for them just down the road from us. Charity Universal is an excellent motto but caring for our neighbour involves us in a rather different way. It is interesting that Fox had a similar problem when building Brislington House. First-class idea, but do it somewhere else. As I mentioned earlier, I had already finished my address when I came across the article on BITHA (Bristol Industrial Therapy Housing Association). The author makes it clear that the problems are not only with the neighbours but also with the local and central authorities. Having cast people out we do not want to have them back unless they can conform in every way.

Again, we all expect a proportion of our patients to relapse, but when delinquents, criminals, offend again, Society (that is us) seldom accepts that it may be, in part, our responsibility. The person having done time has been returned, relatively unchanged, to his old environment equally unchanged and in fact a recurrence may be expected particularly if there is a strong constitutional element.

Our Society has worked through helping people with their physical handicaps, the Welfare State has seen to it that few children are undernourished, we are catching up with educational deficiencies and most adults are literate. We are still, however, struggling when it comes to personal relationships, where the emotional factor rather than the intellectual is all important.

In our culture where the extended family do not live in the same area the advantage of having two parents who compensate for each other's deficiencies cannot be over-stressed. The importance of the early years has long been recognised. 'Give me a child until the age of seven' has always been attributed to the Jesuits, though when I tried to trace it, two Jesuit Scholars said it was 'source unknown'. We know too little about the factors that influence personality development and what decides our view of the environment.

Let me end with a picture (Plate XXXVI) which I think symbolises and illustrates our problem.

**Plate XXXVI**

Which did you see first and why—How long before you saw the second?

## Figures and Tables

**Plate XXXII f1:**
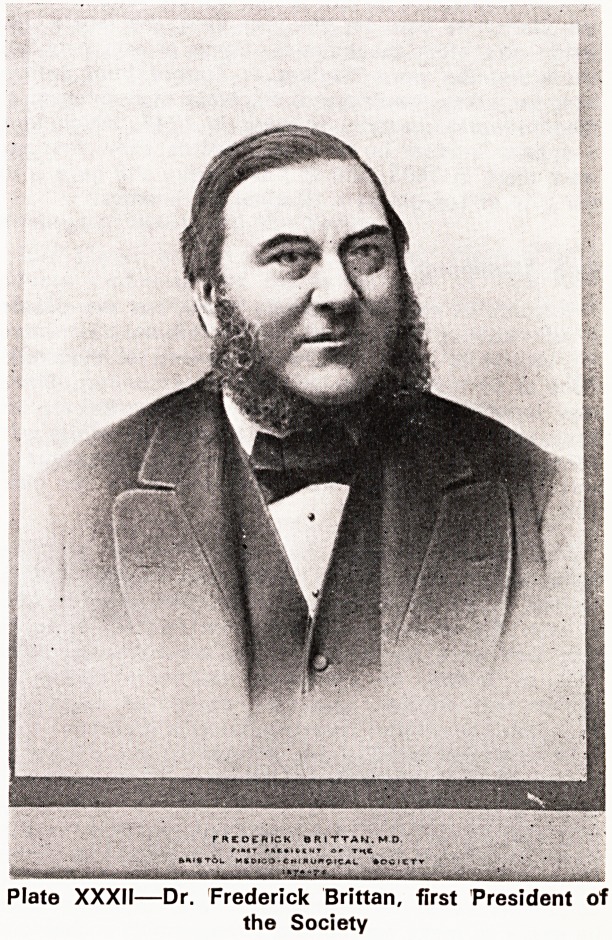


**Plate XXXIII f2:**
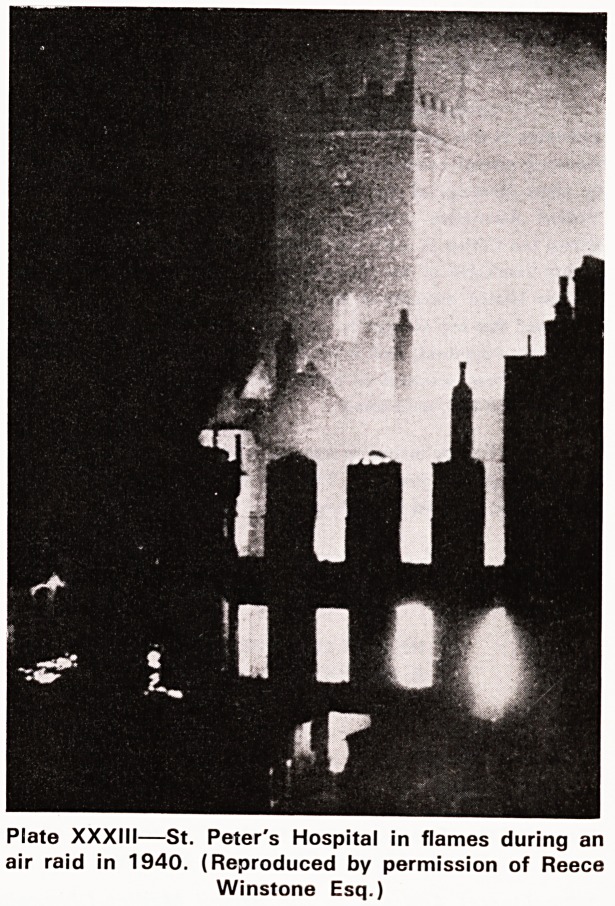


**Plate XXXIV f3:**
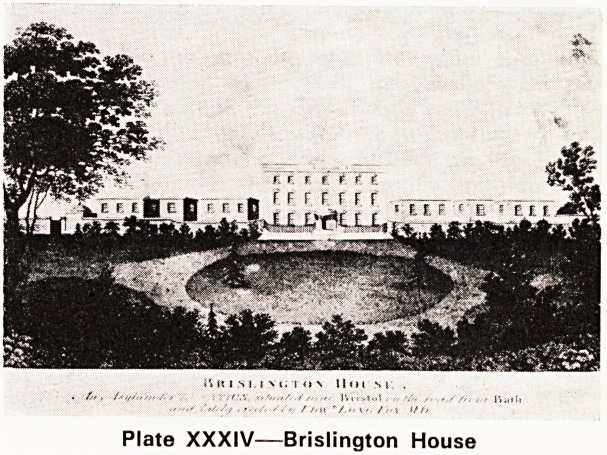


**Plate XXXV f4:**
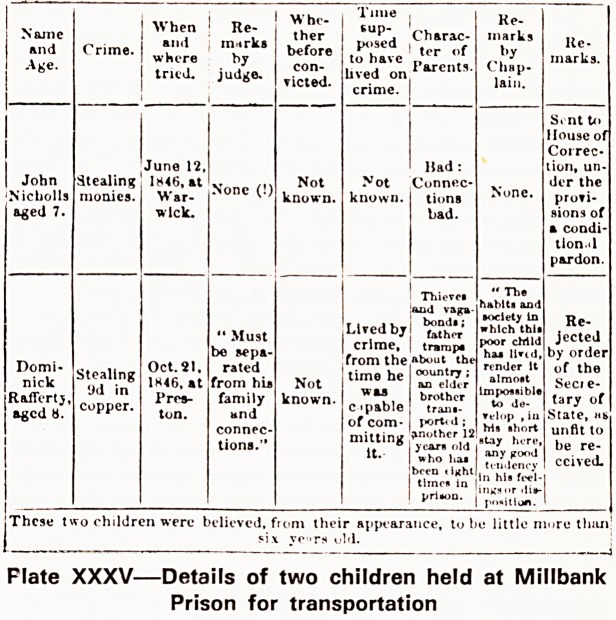


**Fig. 1 f5:**
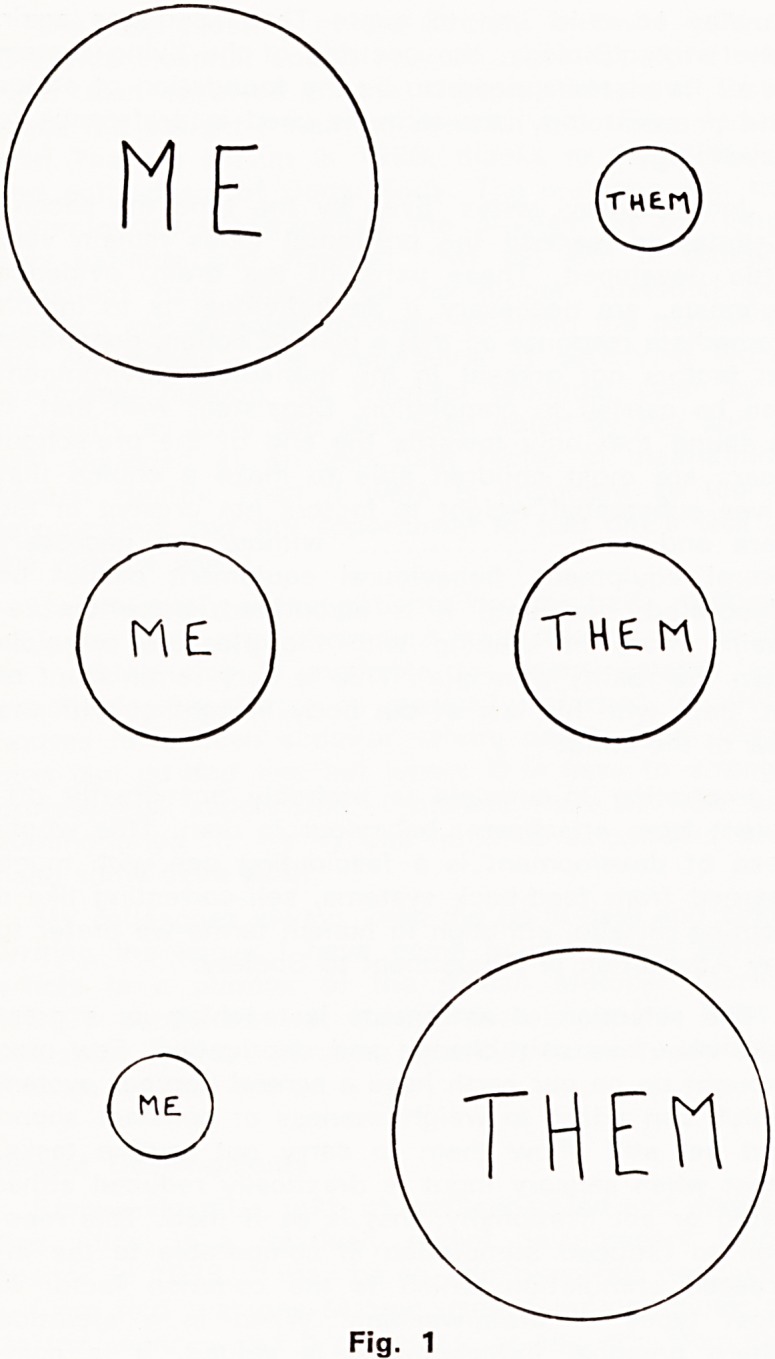


**Plate XXXVI f6:**